# Wearable Sensor Localization Considering Mixed Distributed Sources in Health Monitoring Systems

**DOI:** 10.3390/s16030368

**Published:** 2016-03-12

**Authors:** Liangtian Wan, Guangjie Han, Hao Wang, Lei Shu, Nanxing Feng, Bao Peng

**Affiliations:** 1 Department of Information and Communication Systems, Hohai University, Changzhou 213022, China; wanliangtian1@163.com (L.W.); wanghaohhu@outlook.com (H.W.); 2 Guangdong Petrochemical Equipment Fault Diagnosis Key Laboratory, Guangdong University of Petrochemical Technology, Guangdong 525000, China; lei.shu@ieee.org; 3 Institute of Electromagnetics and Acoustics, Xiamen University, Xiamen 361005, China; fengnaixing@gmail.com; 4 School of Electronic and Communication, Shenzhen Institute of Information Technology, Shenzhen 518172, China; pengb@sziit.com.cn

**Keywords:** health monitoring systems, virtual multiple input and multiple output (VMIMO), direction-of-arrival (DOA) estimation, incoherently-distributed (ID) and coherently-distributed (CD) sources

## Abstract

In health monitoring systems, the base station (BS) and the wearable sensors communicate with each other to construct a virtual multiple input and multiple output (VMIMO) system. In real applications, the signal that the BS received is a distributed source because of the scattering, reflection, diffraction and refraction in the propagation path. In this paper, a 2D direction-of-arrival (DOA) estimation algorithm for incoherently-distributed (ID) and coherently-distributed (CD) sources is proposed based on multiple VMIMO systems. ID and CD sources are separated through the second-order blind identification (SOBI) algorithm. The traditional estimating signal parameters via the rotational invariance technique (ESPRIT)-based algorithm is valid only for one-dimensional (1D) DOA estimation for the ID source. By constructing the signal subspace, two rotational invariant relationships are constructed. Then, we extend the ESPRIT to estimate 2D DOAs for ID sources. For DOA estimation of CD sources, two rational invariance relationships are constructed based on the application of generalized steering vectors (GSVs). Then, the ESPRIT-based algorithm is used for estimating the eigenvalues of two rational invariance matrices, which contain the angular parameters. The expressions of azimuth and elevation for ID and CD sources have closed forms, which means that the spectrum peak searching is avoided. Therefore, compared to the traditional 2D DOA estimation algorithms, the proposed algorithm imposes significantly low computational complexity. The intersecting point of two rays, which come from two different directions measured by two uniform rectangle arrays (URA), can be regarded as the location of the biosensor (wearable sensor). Three BSs adopting the smart antenna (SA) technique cooperate with each other to locate the wearable sensors using the angulation positioning method. Simulation results demonstrate the effectiveness of the proposed algorithm.

## 1. Introduction

Wearable health-monitoring systems (WHMS) have emerged as an effective way of improving the performance of remote diagnoses and patients’ monitoring [1]. As the world population is aging, the healthcare costs will increase, as well. There has been a need to monitor a patient’s status while he or she is out of the hospital in his or her personal environment [2]. Motivated by recent technological advances in microelectronics and wireless communication, the wearable sensors of body area networks (BANs), which are parts of the wireless sensor networks (WSN) [3,4], can be used to locate and monitor patient’s status. Then, the feedback information about patient’s health condition can be provided to caregivers, *i.e.*, medical center, supervising professional physician or even the user himself or herself [5]. The localization of the patient (wearable sensor) is an important parameter for the medical center or caregiver, especially for patients with cardiovascular diseases.

**Figure 1:**
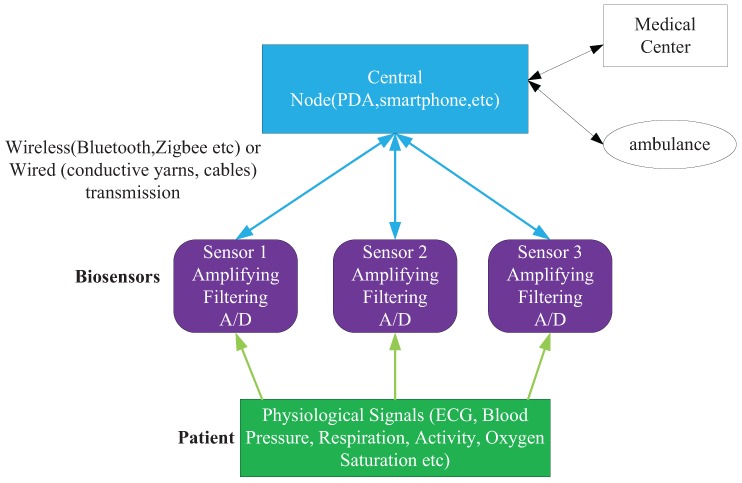
Architecture of a wearable health-monitoring system.

For the 5G link, the peak data rate will likely be in the range of tens of Gbps, which is suitable for real-time communication between wearable sensors and caregivers via a base station (BS), *etc*. [6]. A general WHMS architecture consisting of the system’s functionality and components is depicted in Figure 1. The patients in the hospital or geracomium are relatively intensive. The wearable sensors of patients can be grouped together, and they can communicate with a BS simultaneously on the same resource block. Thus, the BS and the patients construct a virtual multiple input and multiple output (VMIMO) system. At present, the BS mostly adopts a smart antenna (SA) to reduce interference, increase coverage and provide geographic information [7]. The fixed multi-beam antenna and the adaptive array of antennas are two basic types of SA. The former turns the beams on or off while the patient is moving; the latter processes and combines received signals to maximize the signal-to-interference and noise ratio (SINR) [8,9]. The intersecting point of two rays, which come from two different directions measured by two uniform rectangle arrays (URA), can be regarded as the patient’s location. Thus, the accuracy of direction-of-arrival (DOA) estimation [10,11] is crucial in both types of SA.

In real applications, the effect of angular spread cannot be ignored due to the scattering, reflection, diffraction and refraction of the transmitted signal. If the signal model is regarded as a point source, the DOA estimation performance of the VMIMO system [12,13] will degrade significantly. Generally, a spatially-distributed source consists of hundreds of point sources [14]. The distributed source can be categorized into incoherently-distributed (ID) and coherently-distributed (CD) sources corresponding to rapidly and slowly time-varying channels, respectively. For ID source estimation, several DOA estimation algorithms have been proposed in [15,16,17,18]. A new subspace-based algorithm without eigendecomposition of the covariance matrix has been proposed based on the Capon estimator [15] and the property of the inverse of the covariance matrix [16]. However, the 2D nominal DOA of the user terminal (UT) has to be estimated based on spectrum peak searching, and the computational complexity is relatively high [15,16]. The maximum likelihood (ML) estimator, which has the optimal estimation performance, has been proposed in [17,18]. However, only one distributed source can be estimated in [18]. For CD source estimation, many DOA estimation algorithms have been proposed, as well [19,20,21]. The 2D DOA estimation for two distributed source models (parametric and nonparametric) has been solved based on the MUSIC-based method [19]. A two-step procedure enabling decoupling the estimation of DOA from that of the angular spread has been proposed in [20]. For 2D DOA estimation, the sequential one-dimensional searching (SOS) method has been proposed based on uniform circular arrays (UCA) [21]. However, 1D searching is needed, as well.

In this paper, we adopt the angulation positioning method for patients’ (wearable sensors) localizations based on three BSs with the cooperation of multiple VMIMO systems. To the best of our knowledge, there have been few reports about DOA estimation for ID and CD sources jointly. Thus, we propose a 2D DOA estimation algorithm under the coexistence of ID and CD sources. To be more specific, the main contributions of this paper are listed as follows.

(1) Based on the spatiotemporal separation technique, including the second-order blind identification (SOBI) algorithm and the minimum description length (MDL) criterion, the mixed array manifold matrix, including ID and CD sources, is obtained. ID and CD sources can be separated from the amplitude information of the elements of the mixed array manifold matrix.
(2) Based on first-order Taylor series expansion of the steering vector and signal subspace algorithm, we extend the ESPRIT to 2D DOA estimation. Three sub-arrays are constructed to form two rotational invariant relationships, and then, the ESPRIT algorithm is used for DOA estimation of ID sources.
(3) Based on the application of generalized steering vectors (GSVs), two rotational invariant relationships are constructed, as well. We use ESPRIT to estimate eigenvalues of the two rotational invariant matrices. The DOAs of CD sources contained in the eigenvalues can be obtained finally.
(4) Compared to the MUSIC-based method, the proposed algorithm bypasses the spectrum peak searching because of the closed expression of the proposed estimator. Thus, the proposed algorithm has much lower computational complexity, which is suitable for real-time application.
(5) The Cramér-Rao bound (CRB) containing ID and CD sources is derived, whereas the known CRB is only valid for the estimation of CD or ID sources.

This paper is organized as follows. The localization scheme and problem formulation are introduced in Section 2. The source separation algorithm is given in Section 3. The proposed DOA estimation algorithm under the coexistence of ID and CD sources is proposed in Section 4. The simulation results are shown and analyzed in Section 5. The conclusions are drawn in Section 6.

Notation: In this paper, the operator ${\left(\cdot \right)}^{T}$, ${\left(\cdot \right)}^{H}$ and $E\left\{\cdot \right\}$ denote the transpose, conjugate transpose and expectation, respectively. The boldface uppercase letters and boldface lowercase letters denote matrices and column vectors, respectively. The symbol diag$\left\{z_{1},z_{2}\right\}$ stands for a diagonal matrix whose diagonal entries are $z_{1}$ and $z_{2}$. The symbol blkdiag $\left\{Z_{1},Z_{2}\right\}$ stands for a block diagonal matrix, whose diagonal entries are matrices $Z_{1}$ and $Z_{2}$.

## 2. The Localization Scheme Based on VMIMO Systems

### 2.1. The Localization Framework

**Figure 2:**
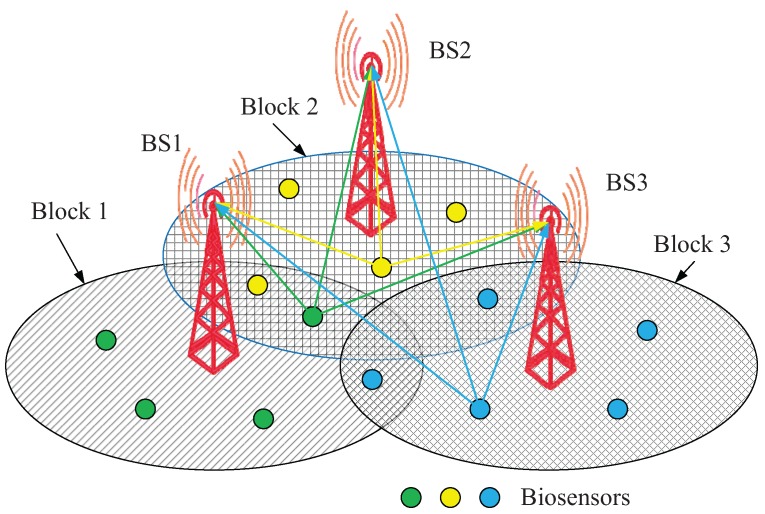
Localization scheme based on VMIMO systems.

The localization scheme of wearable sensors is shown in Figure 2. Three URAs are installed at the top of three BSs, respectively. There are three resource blocks in Figure 2. The green circles (biosensors) and the BS1 construct VMIMO1; the yellow circles (biosensors) and the BS2 construct VMIMO2; the blue circles (biosensors) and the BS3 construct VMIMO3. These biosensors (wearable sensors) can measure significant physiological parameters, like oxygen saturation, heart rate, body and skin temperature, respiration rate, electrocardiogram, *etc*. Generally, the obtained data measured by biosensors are delivered to the BS based on a wireless link via a central node, such as a mobile phone in the same resource block; then, the BS transmits the aggregated vital signs to a medical center or a caregiver. The doctor and caregiver can realize the patient’s physical condition via a user interface, such as a PC, mobile phone, *etc*.

In theory, the intersecting point of two rays, which come from two different directions measured by two uniform rectangle arrays (URA), can be regarded as the patient’s (wearable sensor) location. However, for one biosensor, the BS can only give one estimated result of DOA in one resource block. We need the help of another BS to give another estimated result of DOA. Thus, multiple VMIMO systems (more than two) should cooperate with each other to locate the positions of biosensors. Generally, we use three BSs to locate the positions of biosensors. The third estimated result of BS3 is to guarantee the estimated accuracy and stability. Each biosensor has a unique ID, and the information of all IDs of biosensors has already been stored in the data processing center (DPC). We can match two different rays estimated by two different BSs according to the unique ID. Thus, the position of the biosensor can be located. The DOA estimation algorithm can estimate multiple DOAs simultaneously. Thus, multiple positions of biosensors can be obtained simultaneously. The details can be found in Appendix A1.

### 2.2. The Problem Formulation of DOA Estimation

In VMIMO systems, the rapidly time-varying and slowly time-varying channels corresponding to ID and CD sources both exist. Thus, the mixed distributed sources, which are a more general case, should be considered. In this subsection, the received data model of mixed distributed sources is constructed, and it is adopted in the separation and 2D DOA estimation of ID and CD sources.

**Figure 3:**
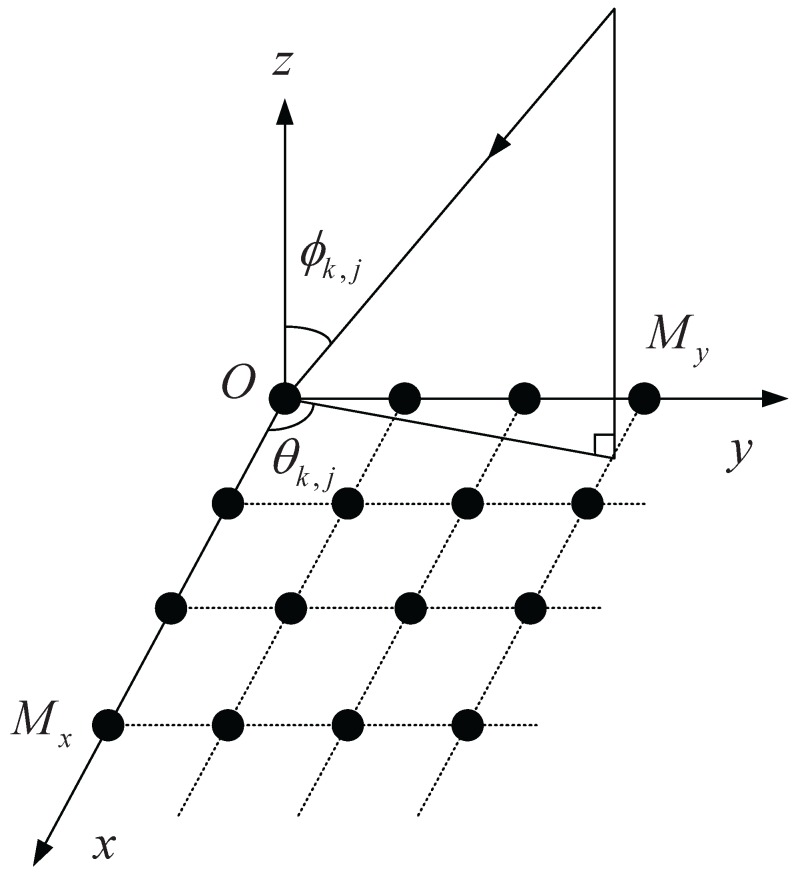
The array configuration of the uniform rectangle array (URA) in a BS.

Assume that *K* narrowband uncorrelated distributed sources impinge on a URA with the number of elements $M=M_{x}M_{y}$, where $M_{x}$ and $M_{y}$ are the number of elements respectively placed along *x* and *y* directions, as shown in Figure 3, *i.e.*, a BS of a VMIMO system with *M* elements communicates with *K* UTs simultaneously using SA technique [8]. The inter-element spacing along the *x* and *y* direction is half the wavelength. In a cellular mobile communication system, due to the reflection and scattering in the multipath propagation, the angular spread cannot be ignored. The point source model is replaced by a distributed source model. Thus, the M×1 received data of a BS can be expressed as [14]:

(1) $$\begin{array}{cc} x\left(t\right) & =\sum_{k=1}^{K_{ID}}s_{k}\left(t\right)\sum_{j=1}^{L_{i}}\gamma_{k,j}\left(t\right)a\left(\theta_{k,j}\left(t\right),\phi_{k,j}\left(t\right)\right)+\\ & \sum_{k=K_{ID}+1}^{K}s_{k}\left(t\right)\int \int a\left(\theta_{k},\phi_{k}\right)\rho_{k}\left(\theta ,\phi ,\mu_{k}\right)d\theta d\phi \\ & =A_{ID}s_{ID}\left(t\right)+A_{CD}s_{CD}\left(t\right)+n\left(t\right)=\mathrm{As}\left(t\right)+n\left(t\right)\in C^{M\times 1} \end{array}$$

where t=1,…,T is a discrete series corresponding to the sampling points from one to *T*, and *T* is the snapshot number. The matrix $A=\left[A_{ID},A_{CD}\right]=\left[h_{1},\ldots ,h_{K_{ID}},h_{K_{ID}+1},\ldots ,h_{K}\right]\in C^{M\times K}$ is the array manifold matrix with the source (biosensor) number $K=K_{ID}+K_{CD}$, where $K_{ID}$ is the source number of ID sources and $K_{CD}$ is the number of CD sources; $h_{k}$ is the steering vector of the *k*-th signal source.

(2) $$\begin{array}{cc} A_{ID}= & \left[\sum_{j=1}^{L_{1}}\gamma_{1,j}\left(t\right)a\left(\theta_{1,j}\left(t\right),\phi_{1,j}\left(t\right)\right)\right.\\ & ,\ldots ,\left.\sum_{j=1}^{L_{K_{ID}}}\gamma_{K_{ID},j}\left(t\right)a\left(\theta_{K_{ID},j}\left(t\right),\phi_{K_{ID},j}\left(t\right)\right)\right]\in C^{M\times K_{ID}} \end{array}$$

and:

(3) $$\begin{array}{cc} A_{CD}= & \left[\int \int a\left(\theta_{K_{ID}+1},\phi_{K_{ID}+1}\right)\rho_{1}\left(\theta ,\phi ,\mu_{K_{ID}+1}\right)d\theta d\phi \right.\\ & \left.,\ldots ,\int \int a\left(\theta_{K},\phi_{K}\right)\rho_{K}\left(\theta ,\phi ,\mu_{K}\right)d\theta d\phi \right]\in C^{M\times K_{CD}} \end{array}$$

are the array manifold matrices corresponding to ID and CD sources, respectively. The signals vector $s\left(t\right)={\left[s_{ID}^{T}\left(t\right),s_{CD}^{T}\left(t\right)\right]}^{T}\in C^{K\times 1}$ is the signal transmitted by biosensors. $s_{ID}\left(t\right)=\left[s_{1}\left(t\right),\ldots ,s_{K_{ID}}\left(t\right)\right]\in C^{K_{ID}\times 1}$ and $s_{CD}\left(t\right)=\left[s_{K_{ID}+1}\left(t\right),\ldots ,s_{K}\left(t\right)\right]\in C^{K_{CD}\times 1}$ are the signal vectors corresponding to ID and CD sources, respectively. $\gamma_{k,j}\left(t\right)$, $\theta_{k,j}\left(t\right)$ and $\phi_{k,j}\left(t\right)$ are the complex gain, azimuth and elevation of the *j*-th path for the *k*-th ID source, respectively, which satisfy $0\leq \theta_{k,j}\left(t\right)\leq \;\pi$, 0≤ϕk,jt≤ππ22, $k=1,\ldots ,K_{ID}$, $j=1,\ldots ,L_{k}$. $L_{k}$ is the number of multipaths of the *k*-th ID source.

The steering vector $a\left(\theta_{k,j}\left(t\right),\phi_{k,j}\left(t\right)\right)\in C^{M\times 1}$ is the array response corresponding to $\theta_{k,j}\left(t\right)$ and $\phi_{k,j}\left(t\right)$. The *m*-th element of the array is given by:

(4) $$\begin{array}{cc} {\left[a\left(\theta_{k,j}\left(t\right),\phi_{k,j}\left(t\right)\right)\right]}_{m}= & \exp\left(i\omega \sin\left(\phi_{k,j}\left(t\right)\right)\right.\left[\left(m_{x}-1\right)\right.\\ & \times \left.\left.\cos\left(\theta_{k,j}\left(t\right)\right)+\left(m_{y}-1\right)\sin\left(\theta_{k,j}\left(t\right)\right)\right]\right),\\ & m=\left(m_{y}-1\right)+M_{x},m_{x}=1,\ldots ,M_{x},m_{y}=1,\ldots ,M_{y} \end{array}$$

where ω=2πd2πdλλ, *d* and *λ* are the inter-sensor spacing and the wavelength, respectively. The azimuth and elevation can be respectively written in another form as:

(5) $$\theta_{k,j}\left(t\right)={\bar{\theta }}_{k}+{\tilde{\theta }}_{k,j}\left(t\right)$$

(6) $$\phi_{k,j}\left(t\right)={\bar{\phi }}_{k}+{\tilde{\phi }}_{k,j}\left(t\right)$$

where ${\bar{\theta }}_{k}$ and ${\bar{\phi }}_{k}$ are nominal azimuth and elevation of the *k*-th ID source, respectively, ${\tilde{\theta }}_{k,j}\left(t\right)$ and ${\tilde{\phi }}_{k,j}\left(t\right)$ are referred to as the angular spreads, which correspond to random angular deviations with zero mean and standard deviations $\sigma_{\theta_{k}}$ and $\sigma_{\phi_{k}}$, respectively. $\rho_{i}\left(\theta ,\phi ,\mu_{i}\right)$ is a deterministic angular distribution function of the *k*-th ID source, $k=K_{ID}+1,\ldots ,K$. The parameterized vector $\mu_{i}$ is expressed as $\mu_{i}=\left(\theta_{i},\sigma_{\theta_{i}},\phi_{i},\sigma_{\phi_{i}}\right)$. $n\left(t\right)\in C^{M\times 1}$ is the additive noise.

In addition, some assumptions are considered throughout this paper.

(1) ID sources are uncorrelated with CD sources, and they are uncorrelated with the noise.
(2) The complex gains $\gamma_{k,j}\left(t\right)$, $k=1,\ldots ,K_{ID}$, $j=1,\ldots ,L_{k}$, t=1,…,T, are independent and identically distributed (i.i.d.) complex-valued zero-mean random variables:
  (7) $$E\left\{\gamma_{k,j}\left(t\right)\gamma_{k,j}^{*}\left(\tilde{t}\right)\right\}=\frac{\sigma_{\gamma_{k}}^{2}}{L_{k}}\delta \left(k-\tilde{k}\right)\delta \left(j-\tilde{j}\right)\delta \left(t-\tilde{t}\right)$$
  where $E\left\{\gamma_{k,j}\left(t\right)\gamma_{k,j}^{*}\left(\tilde{t}\right)\right\}$ is the covariance.
(3) The noise $n\left(t\right)$, t=1,…,T is an i.i.d. random variable both in temporally- and spatially-complex-valued circularly symmetric zero-mean Gaussian variables, whose covariance matrix is given by:
  (8) $$E\left\{n\left(t\right)n^{H}\left(\tilde{t}\right)\right\}=\sigma_{n}^{2}I_{M}\delta \left(t-\tilde{t}\right)$$
  where $E\left\{n\left(t\right)n^{H}\left(\tilde{t}\right)\right\}$ is the covariance matrix.
(4) The number of multipaths $L_{k}$ is sufficiently large, $k=1,\ldots ,K_{ID}$.
(5) The BS equipped with *M* elements is much larger than the number of biosensors *K* [22].
(6) The signal powers $S_{IDk}={\left|s_{IDk}\left(t\right)\right|}^{2}$, $k=1,\ldots ,K_{ID}$ of ID sources are known as a prior; the cellular area of the same resource block is not large; thus the power loss can be ignored.

Finally, we emphasize that the task is to estimate 2D nominal DOA ${\bar{\theta }}_{k}$ and ${\bar{\phi }}_{k}$ for ID and CD sources, respectively, k=1,…,K, based on the received snapshot data $x\left(t\right)$, t=1,…,T.

## 3. The Distributed Source Classification Based on SOBI

In this section, we will separate the ID and CD sources from the received signals with the help of the SOBI algorithm [23,24].

The covariance matrix of the *k*-th ID source can be modeled as [25]:

(9) $$R_{IDk}\approx S_{IDk}a_{k}\left(\theta ,\phi \right)a_{k}^{H}\left(\theta ,\phi \right)⊙B\left(\Phi_{k}\right)$$

where $B\left(\Phi_{k}\right)$ is the real-valued symmetric Toeplitz matrix (the derivation is given in Appendix A2).

The channel of the *k*-th CD source is defined as [26]:

(10) $$h_{CDk}=\int \int a\left(\theta_{k},\phi_{k}\right)\rho_{k}\left(\theta ,\phi ,\mu_{k}\right)d\theta d\phi \approx a\left(\theta_{k},\phi_{k}\right)⊙g\left(\mu_{k}\right),k=K_{ID}+1,\ldots ,K$$

where $g\left(\mu_{k}\right)$ is a real-valued term.

The SOBI algorithm is a blind signal processing technique based on a second-order statistic characteristic. For the *k*-th source $s_{k}\left(t\right)$, the covariance matrix with nonzero time lag *e* can be expressed as [23]:

(11) $$f_{k}=E\left\{s_{k}\left(t\right)s_{k}^{*}\left(t-e\right)\right\}$$

Then, the correlation covariance of $x\left(t\right)$ with time lag *e* is given by:

(12) $$R\left(e\right)=E\left\{x\left(t\right)x^{H}\left(t-e\right)\right\}=\sum_{k=1}^{K}f_{k}\left(e\right)h_{k}h_{k}^{H}=AD_{e}A^{H}$$

where $D_{e}=\text{diag}\left(\left[f_{1}\left(e\right),\ldots ,f_{K}\left(e\right)\right]\right)$. Note that $R\left(e_{l}\right)$, l=1,…,L contain the identical array manifold with respect to different $e\in \left\{e_{1},\ldots ,e_{L}\right\}$. It can be proven that if the difference among the diagonal entries of $D_{e}$ is obvious and **A** is full column rank, then multiple $R\left(e_{l}\right)$ can be joint diagonalized using **A** or its permutation matrix. Then, the array manifold matrix **A** can be accurately estimated using $R\left(e_{l}\right)$ with different time lag *e*. Assume that there are two correlation covariance matrices $R\left(e_{1}\right)$ and $R\left(e_{2}\right)$; we have:

(13) $$R\left(e_{1}\right)R^{-1}\left(e_{2}\right)=AD_{e_{1}}D_{e_{2}}^{-1}A^{H}$$

Because $D_{e_{1}}D_{e_{2}}^{-1}$ is a diagonal matrix, the unique estimate of array manifold matrix **A** can be obtained by taking the eigenvalue decomposition (EVD) of $D_{e_{1}}D_{e_{2}}^{-1}$.

In real applications, $R\left(e\right)$ can be estimated by the limited sample, which is given by:

(14) $$\hat{R}\left(e_{l}\right)=\frac{1}{T}\sum_{i=p+1}^{T+p+1}E\left\{x\left(i\right)x^{H}\left(i-l\right)\right\},l=0,\ldots ,L,p=1,\ldots ,P$$

Then, the SOBI algorithm is summarized as Algorithm 1 [24].
**Algorithm 1** Implementation of the SOBI Algorithm.
1:Estimate the sampling covariance matrix $\hat{R}\left(0\right)$ using *T* snapshot number;2:Take EVD of $\hat{R}\left(0\right)$; $\lambda_{1},\ldots ,\lambda_{n}$ are *n* large eigenvalues, and $u_{1},\ldots ,u_{n}$ are their corresponding eigenvectors;3:Average M−n small eigenvalues of $\hat{R}\left(0\right)$, the noise variance ${\hat{\sigma }}_{n}^{2}$ can be obtained; the whitened signal $z\left(t\right)=\left[z_{1}\left(t\right),\ldots ,z_{n}\left(t\right)\right]$ can be obtained with $z_{i}\left(t\right)=\left(\lambda_{i}-{\hat{\sigma }}_{n}^{2}\right)u_{i}^{*}x\left(t\right)$. It is equivalent to construct a whitened matrix $\hat{W}={\left[{\left(\lambda_{1}-{\hat{\sigma }}_{n}^{2}\right)}^{-\left(1/2\right)}u_{1},\ldots ,{\left(\lambda_{1}-{\hat{\sigma }}_{n}^{2}\right)}^{-\left(1/2\right)}u_{n}\right]}^{H}$;4:Calculate the sampling covariance matrix $\hat{R}\left(e_{l}\right)$ of $z\left(t\right)$ with fixed time lag $e\in \left\{e_{1},\ldots ,e_{L}\right\}$, *L* is the group number of the sampling covariance matrix;5:Take the joint diagonalization of $\hat{R}\left(e_{1}\right),\ldots ,\hat{R}\left(e_{L}\right)$ as shown in (13), then a unitary matrix $\hat{U}$ is obtained by taking the EVD of result matrix of joint diagonalization;6:Estimate the generalized array manifold matrix **A** using $\hat{A}={\hat{W}}^{†}\hat{U}$.


As shown in Equation (9), for the ID source, the amplitude of the entries of the covariance matrix are getting smaller as angular spreads increase; as shown in Equation (10), the amplitude of entries of generalized steering vectors are getting smaller as angular spreads increase.

For distributed sources, the amplitude of entries of array manifold matrix **A** is usually less than one. However, if *n* (source number (SN)) is larger than the actual source number, after the signals’ separation, the amplitude of entries of array manifold matrix **A** is usually lager than one based on experiment. When SN is equal to the actual source number, the amplitude of entries of array manifold matrix **A** is usually not larger than one. Usually, only the ID source may cause this phenomenon. If the ID sources exist, SN is larger than the actual source number based on the minimum description length (MDL) criterion [27]. Thus, the amplitude of any entries of array manifold matrix **A** is larger than one, and SN > 1; we can distinguish it as an ID source. Then, SN is reduced by one, and the SOBI is applied again. This procedure can be done iteratively until all ID sources are separated. Based on the discussion above and Algorithm 1 , the separation algorithm for ID and CD sources is summarized as Algorithm 2.
**Algorithm 2** The Separation Algorithm for ID and CD Sources.1:initialize RSN=0,2:ESN=0;3:SN=n;4:**while**
${\hat{A}}_{i}\left(1:ESN\right)>1$
**do**5:  **if**
${\hat{A}}_{i}>1$
**then**6:   ${\hat{A}}_{ID}=\left[{\hat{A}}_{i},{\hat{A}}_{ID}\right]$;7:   RSN=RSN+1;8:   ESN=SN−RSN;9:   n=n−110:   **Repeat**: **Algorithm** 1;11:  **end if**12:**end while**;
  **return**
*RSN*;
  **return**
*RSN*.


## 4. The Proposed Algorithm

### 4.1. DOA Estimation for ID Source

For the VMIMO system, the existing subspace-based and covariance matching-based algorithms are sophisticated for 2D DOA estimation, because of their tremendous computational complexity of multidimensional searching. In this section, based on the array manifold matrix estimated in the previous section, an ESPRIT-based algorithm is proposed to deal with the problem of 2D DOA estimation with low computational complexity.

Based on first-order Taylor series expansion of $a\left(\theta_{k,j}\left(t\right),\phi_{k,j}\left(t\right)\right)$, the steering vector in Equation (4) can be approximated as:

(15) $$\begin{array}{cc} a\left(\theta_{k,j}\left(t\right),\phi_{k,j}\left(t\right)\right) & =a\left({\bar{\theta }}_{k}+{\tilde{\theta }}_{k,j}\left(t\right),{\bar{\phi }}_{k}+{\tilde{\phi }}_{k,j}\left(t\right)\right)\\ & \approx a\left({\bar{\theta }}_{k},{\bar{\phi }}_{k}\right)+\frac{\partial a\left({\bar{\theta }}_{k},{\bar{\phi }}_{k}\right)}{\partial {\bar{\theta }}_{k}}{\tilde{\theta }}_{k,j}\left(t\right)+\frac{\partial a\left({\bar{\theta }}_{k},{\bar{\phi }}_{k}\right)}{\partial {\bar{\phi }}_{k}}{\tilde{\phi }}_{k,j}\left(t\right) \end{array}$$

where the terms after the second term are ignored. If standard deviations $\sigma_{\theta_{k}}$ and $\sigma_{\phi_{k}}$ are sufficiently small, the first-order Taylor series expansion is almost equal to $a\left(\theta_{k,j}\left(t\right),\phi_{k,j}\left(t\right)\right)$. Then, the received data of ID source can be rewritten as:

(16) $$x_{ID}\left(t\right)\approx \sum_{k=1}^{K_{ID}}\left(a\left({\bar{\theta }}_{k},{\bar{\phi }}_{k}\right)c_{k,1}\left(t\right)+\frac{\partial a\left({\bar{\theta }}_{k},{\bar{\phi }}_{k}\right)}{\partial {\bar{\theta }}_{k}}c_{k,2}\left(t\right)\left.+\frac{\partial a\left({\bar{\theta }}_{k},{\bar{\phi }}_{k}\right)}{\partial {\bar{\phi }}_{k}}c_{k,3}\left(t\right)\right)+n\left(t\right)\in C^{M\times 1}\right.$$

where $c_{k,1}\left(t\right)=s_{k}\left(t\right)\sum_{j=1}^{N_{k}}D_{k,j}\left(t\right)$, $c_{k,2}\left(t\right)=s_{k}\left(t\right)\sum_{j=1}^{N_{k}}D_{k,j}\left(t\right){\tilde{\theta }}_{k,j}\left(t\right)$ and $c_{k,3}\left(t\right)=s_{k}\left(t\right)\sum_{j=1}^{N_{k}}D_{k,j}\left(t\right){\tilde{\phi }}_{k,j}\left(t\right),k=1,\ldots ,K_{ID}$.

It can be seen from Equation (16) that the relationship between the received data of the ID source and $a\left(\theta_{k,j}\left(t\right),\phi_{k,j}\left(t\right)\right)$ is linear; so is its partial derivatives. Thus, the received data of ID sources can be rewritten as:

(17) $$x_{ID}\left(t\right)\approx B_{ID}\left(t\right)c\left(t\right)+n\left(t\right)$$

where:

(18) $$\begin{array}{cc} B_{ID}\left(t\right)= & \left[a\left({\bar{\theta }}_{1},{\bar{\phi }}_{1}\right),\ldots ,a\left({\bar{\theta }}_{K_{ID}},{\bar{\phi }}_{K_{ID}}\right)\right.,\frac{\partial a\left({\bar{\theta }}_{1},{\bar{\phi }}_{1}\right)}{\partial {\bar{\theta }}_{1}}\\ & ,\ldots ,\frac{\partial a\left({\bar{\theta }}_{K_{ID}},{\bar{\phi }}_{K_{ID}}\right)}{\partial {\bar{\theta }}_{K_{ID}}},\left.\frac{\partial a\left({\bar{\theta }}_{1},{\bar{\phi }}_{1}\right)}{\partial {\bar{\phi }}_{1}},\ldots ,\frac{\partial a\left({\bar{\theta }}_{K_{ID}},{\bar{\phi }}_{K_{ID}}\right)}{\partial {\bar{\phi }}_{K_{ID}}}\right]\in C^{M\times 3K_{ID}} \end{array}$$

Then, the new array manifold matrix is expressed as:

(19) $$c\left(t\right)=\left[c_{1,1}\left(t\right),\ldots ,c_{K_{ID},1}\left(t\right),c_{1,2}\left(t\right),\ldots ,\left.c_{K_{ID},2}\left(t\right),\ldots ,c_{1,3}\left(t\right),\ldots ,c_{K_{ID},3}\left(t\right)\right]\in C^{3K_{ID}\times 1}\right.$$

the elements of c(t) are functions of the incident signal, the path gains and angular deviations. It can be known that A is only determined by the nominal DOAs, ${\bar{\theta }}_{k}$ and ${\bar{\phi }}_{k}$, $k=1,\ldots ,K_{ID}$. Thus, we can obtain the DOA of ID sources from A, and the covariance matrix of $c\left(t\right)$ can be expressed as:

(20) $$P_{c}=E\left\{c\left(t\right)c^{H}\left(t\right)\right\}\in R^{3K_{ID}\times 3K_{ID}}$$

It is a diagonal matrix with $S_{IDk}={\left|s_{IDk}\left(t\right)\right|}^{2}$, ${\left[P_{c}\right]}_{K+k,K+k}={\left[P_{c}\right]}_{k,k}\sigma_{\theta_{k}}^{2}$, ${\left[P_{c}\right]}_{2K+k,2K+k}={\left[P_{c}\right]}_{k,k}\sigma_{\phi_{k}}^{2}$, $k=1,\ldots ,K_{ID}$.

Based on Algorithm 2, the steering vectors of ID sources are separated. The estimator of ${\hat{A}}_{ID}$ is obtained. Then, the estimator of snapshot data ${\hat{x}}_{ID}\left(t\right)$ for ID sources can be obtained by:

(21) $${\hat{x}}_{ID}\left(t\right)\approx {\hat{A}}_{ID}s\left(t\right)$$

Then, the covariance matrix of ${\hat{A}}_{ID}$ is expressed as:

(22) $${\hat{R}}_{ID}=E\left\{{\hat{x}}_{ID}\left(t\right){\hat{x}}_{ID}^{H}\left(t\right)\right\}={\hat{A}}_{ID}{\hat{R}}_{IDs}{\hat{A}}_{ID}^{H}\approx B_{ID}P_{c}B_{ID}^{H}+\sigma_{n}^{2}I_{M}\in C^{M\times M}$$

where ${\hat{R}}_{IDs}=\text{diag}\left\{S_{ID1},\ldots ,S_{IDK_{ID}}\right\}$. It can be known that ${\hat{R}}_{IDs}$ is a normal and positive diagonal matrix. In general, $B_{ID}$ is a full column rank matrix; the EVD of ${\hat{R}}_{ID}$ is given by:

(23) $$\begin{array}{cc} {\hat{R}}_{ID} & \approx \left[U_{s},U_{n}\right]\left[\begin{array}{cc} Q_{s} & 0_{3K_{ID}\times \left(M-3K_{ID}\right)}\\ 0_{\left(M-3K_{ID}\right)\times 3K_{ID}} & \sigma_{n}^{2}I_{M-3K_{ID}} \end{array}\right]{\left[U_{s},U_{n}\right]}^{H}\\ & =U_{s}Q_{s}U_{s}^{H}+\sigma_{n}^{2}U_{n}U_{n}^{H}\approx U_{s}{\tilde{Q}}_{s}U_{s}^{H}+\sigma_{n}^{2}I_{M}, \end{array}$$

where $Q_{s}\in C^{3K_{ID}\times 3K_{ID}}$ is a diagonal matrix with the entries of $3K_{ID}$ large eigenvalues of ${\hat{R}}_{ID}$. The remaining $M-3K_{ID}$ small eigenvalues of ${\hat{R}}_{ID}$ equal to $\sigma_{n}^{2}$. Their corresponding subspace are signal subspace $U_{s}\in C^{M\times 3K_{ID}}$ and noise subspace $U_{n}\in C^{M\times \left(M-3K_{ID}\right)}$, respectively. ${\tilde{Q}}_{s}=Q_{s}-\sigma_{n}^{2}I_{3K}\in R^{3K_{ID}\times 3K_{ID}}$. Based on Equations (22) and (23), we can know that:

(24) $$B_{ID}P_{c}B_{ID}^{H}\approx U_{s}{\tilde{Q}}_{s}U_{s}^{H}$$

Since ${\tilde{Q}}_{s}$ is a diagonal matrix, as well, there exists a nonsingular matrix $T\in C^{3K_{ID}\times 3K_{ID}}$, which satisfies:

(25) $$B_{ID}\approx U_{s}T$$

The linear relationship between $B_{ID}$ and $U_{s}$ will be used to estimate the nominal DOAs.

**Figure 4:**
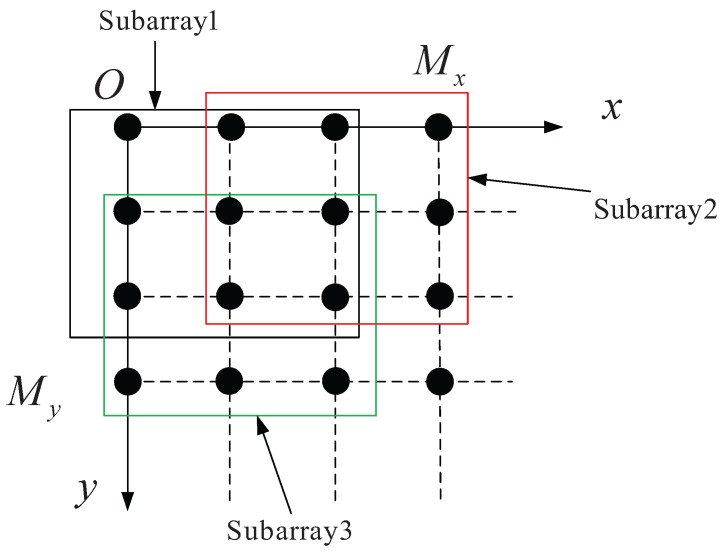
The sub-arrays of URA.

In order to obtain 2D angle estimation, azimuth and elevation, two rotation-invariant relationships have to be constructed [28]. As shown in Figure 4, the whole URA is divided into three sub-arrays. Although more sub-arrays can be divided, the computation complexity increases rapidly in the VMIMO system. In order to reduce the computational complexity, only two rotation-invariant relationships are adopted. This is different from the conventional ESPRIT algorithm; this rotational-invariant relationship contains $a\left({\bar{\theta }}_{k},{\bar{\phi }}_{k}\right)$ and its partial derivatives. The array manifold matrices of sub-arrays have the same form as that of URA. The steering vector of the *l*-th sub-array is defined as $a_{l}\left({\bar{\theta }}_{k},{\bar{\phi }}_{k}\right)\in C^{\tilde{M}\times 1}$, l=1,2,3 and $\tilde{M}=\left(M_{x}-1\right)\left(M_{y}-1\right)$.

In order to estimate the subspaces $U_{ID1}$, $U_{ID2}$ and $U_{ID3}$ of three sub-arrays, we need to construct a selection matrix as follows:

(26) $${\left[J_{l}\right]}_{m,n}=\left\{\begin{array}{c} 1,n=m+\left\lfloor \frac{m-1}{M_{x}-1}\right\rfloor +d_{l},m=1,\ldots ,\tilde{M}\\ 0,otherwise \end{array}\right.$$

where $d_{1}=0$, $d_{2}=1$ and $d_{3}=M_{x}$. In (26), the floor operator makes m−1m−1Mx−1Mx−1=n, $\forall n=0,\ldots ,M_{y}-2$ when $m=n\left(M_{x}-1\right)+1,\ldots ,\left(n+1\right)\left(M_{x}-1\right)$. It can be seen that for the *m*-th row of $J_{l}$, only the m+m−1m−1Mx−1Mx−1+dl-th entry is one, and the other entries are zeros. Thus, $J_{l}$ assigns the m+m−1m−1Mx−1Mx−1+dl-th of $U_{s}$ into the subspaces $U_{ID1}$, $U_{ID2}$ and $U_{ID3}$ belonging to three sub-arrays, respectively. This coincides with the relationships between the sub-arrays and the URA. Then, the estimators of subspaces $U_{ID1}$, $U_{ID2}$ and $U_{ID3}$ can be expressed as:

(27) $$U_{IDl}=J_{l}U_{s},l=1,2,3$$

**Proposition** **1.**  *The subspaces* $U_{ID1}$, $U_{ID2}$ *and* $U_{ID3}$ *belonging to three sub-arrays have the relationships as follows:*

(28) $$\begin{array}{c} U_{ID1}V_{1}=U_{ID2}\\ U_{ID1}V_{2}=U_{ID3} \end{array}$$

*where:*

(29) $$\begin{array}{c} V_{1}=TW_{2,1}T^{-1}\in C^{3K_{ID}\times 3K_{ID}}\\ V_{2}=TW_{3,1}T^{-1}\in C^{3K_{ID}\times 3K_{ID}} \end{array}$$

**Proof.**  See Appendix A3. ☐

Thus, eigenvalues of $V_{1}$ and $V_{2}$ are diagonal entries of $W_{2,1}$ and $W_{3,1}$, respectively. However, $W_{2,1}$ and $W_{3,1}$ are not diagonal matrices, but upper triangular matrices. Then, the conventional ESPRIT algorithm cannot be used directly. Therefore, in order to estimate the diagonal elements of $W_{2,1}$ and $W_{3,1}$, $V_{1}$ and $V_{2}$ need to be estimated from the three subspaces $U_{ID1}$, $U_{ID2}$ and $U_{ID3}$. According to Equation (28), the estimators of $V_{1}$ and $V_{2}$ can be obtained by employing the well-known total least-squares (TLS) method. Referring to the similar derivation in [29], we construct a new matrix:

(30) $${\tilde{U}}_{ID1}\overset{\Delta }{\;=}\left[U_{ID1},U_{ID2}\right]\in C^{\tilde{M}\times 6K_{ID}}$$

and the rank of ${\tilde{U}}_{ID1}$ is $6K_{ID}$. The EVD of ${\tilde{U}}_{ID1}$ is expressed as:

(31) $${\left[U_{ID1},U_{ID2}\right]}^{H}\left[U_{ID1},U_{ID2}\right]=U_{x}P_{x}U_{x}^{H}\in C^{6K_{ID}\times 6K_{ID}}$$

where $U_{x}\in C^{6K_{ID}\times 6K_{ID}}$ and $P_{x}\in C^{6K_{ID}\times 6K_{ID}}$ are eigenvectors and eigenvalues of ${\tilde{U}}_{ID1}$, respectively. Then, $U_{x}\in C^{6K_{ID}\times 6K_{ID}}$ can be partitioned into four block matrices as:

(32) $$U_{x}=\left[\begin{array}{cc} U_{x11} & U_{x12}\\ U_{x21} & U_{x22} \end{array}\right]$$

where $U_{xab}\in C^{3K_{ID}\times 3K_{ID}}$, a,b=1,2. The estimator of $V_{1}$ is expressed as:

(33) $${\hat{V}}_{1}=-U_{x12}U_{x22}^{-1}\in C^{3K_{ID}\times 3K_{ID}}$$

In order to estimate nominal DOAs, the EVD of ${\hat{V}}_{1}$ is expressed as:

(34) $${\hat{V}}_{1}=T_{1}P_{1}T_{1}^{H}$$

where $T_{1}\in C^{3K_{ID}\times 3K_{ID}}$ and $P_{1}\in C^{3K_{ID}\times 3K_{ID}}$ are eigenvectors and eigenvalues of ${\hat{V}}_{1}$, respectively. Similar to the process of estimating ${\hat{V}}_{1}$, the estimator of $V_{2}$ is expressed as:

(35) $${\hat{V}}_{2}=T_{2}P_{2}T_{2}^{H}$$

where $T_{2}\in C^{3K_{ID}\times 3K_{ID}}$ and $P_{2}\in C^{3K_{ID}\times 3K_{ID}}$ are the eigenvectors and eigenvalues of ${\hat{V}}_{2}$, respectively. However, the EVDs of ${\hat{V}}_{1}$ and ${\hat{V}}_{2}$ are completed separately. The eigenvalues between $P_{1}$ and $P_{2}$ have to be matched. Denote the *k*-th diagonal entry of $P_{d}$ as $P_{dk}$, d=1,2. The eigenvectors of the identical source are strongly correlated; thus, we can construct the sequencing matrix $G=T_{1}^{H}T_{2}$ to match $P_{1k}$ and $P_{2i}$. According to the coordinate of the maximal entry in the columns (or rows) of matrix G, we adjust the order of eigenvectors. Then, the parameter pairing is completed. The estimators of ${\left[W_{2,1}\right]}_{k+\left(l-1\right)K,k+\left(l-1\right)K}$ and ${\left[W_{3,1}\right]}_{k+\left(l-1\right)K,k+\left(l-1\right)K}$, which are the eigenvalues $P_{1,3\left(k-1\right)+l}$ and $P_{2,3\left(k-1\right)+l}$, l=1,2,3 of ${\hat{V}}_{1}$ and ${\hat{V}}_{2}$, respectively, are obtained. According to Equations (34) and (35), we have:

(36) $$\lambda_{1,3\left(k-1\right)+l}\approx \exp\left(i\omega \sin\left({\bar{\phi }}_{k}\right)\cos\left({\bar{\theta }}_{k}\right)\right)$$

(37) $$\lambda_{2,3\left(k-1\right)+l}\approx \exp\left(i\omega \sin\left({\bar{\phi }}_{k}\right)\sin\left({\bar{\theta }}_{k}\right)\right)$$

The estimators of the nominal 2D DOAs ${\bar{\theta }}_{k}$ and ${\bar{\phi }}_{k}$ for ID sources are given by:

(38) $${\hat{\bar{\theta }}}_{k}=\frac{1}{3}\sum_{l=1}^{3}\arctan\left(\frac{\lambda_{2,3\left(k-1\right)+l}}{\lambda_{1,3\left(k-1\right)+l}}\right)$$

(39) $${\hat{\bar{\phi }}}_{k}=\frac{1}{3}\sum_{l=1}^{3}\arcsin\left(\frac{1}{\omega }\sqrt{-\sum_{a=1}^{2}{\left(\ln\left(\lambda_{a,3\left(k-1\right)+l}\right)\right)}^{2}}\right)$$

where $k=1,\ldots ,K_{ID}$. Thus, the 2D DOA estimation for ID sources is completed. The pseudo-code is summarized as Algorithm 3.
**Algorithm 3** The DOA Estimation Algorithm for ID Sources.
1:Estimate the covariance matrix ${\hat{R}}_{ID}$ according to Equation (25);2:Take the EVD of ${\hat{R}}_{ID}$; $Q_{s}$ is a diagonal matrix with the entries of $3K_{ID}$; large eigenvalues of ${\hat{R}}_{ID}$, $U_{s}\in C^{M\times 3K_{ID}}$ are the corresponding signal subspace;3:Divide the URA into three sub-arrays; calculate the selection matrix according to Equations (A5)–(A7); construct two different rotational invariant relationships according to Equation (46);4:Construct new matrix ${\tilde{U}}_{ID1}$; perform the EVD of Equation (31), partitioning the matrix $U_{x}$ into four blocks; the eigenvalues of ${\hat{V}}_{1}$ and ${\hat{V}}_{2}$ can be obtained according to Equation (33);5:Estimate 2D DOA for ID sources according to Equations (38) and (39).


### 4.2. DOA Estimation for the CD Source

We first define three $\tilde{M}\times M$ selection matrices:

(40) $$\begin{array}{c} S_{1}=\left[{\tilde{J}}_{0},0_{\tilde{M}\times M}\right]\\ S_{2}=\left[0_{\tilde{M}\times M},{\tilde{J}}_{0}\right]\\ S_{3}=\left[{\tilde{J}}_{1},0_{\tilde{M}\times M}\right] \end{array}$$

where ${\tilde{J}}_{i}=\mathrm{blkdiag}\left\{J_{M\times i},\ldots ,J_{M\times i}\right\}$, i=0,1. The received data of the three sub-arrays for CD sources can be expressed as:

(41) $$\begin{array}{c} x_{CD1}\left(t\right)=\sum_{k=1}^{K_{CD}}\int \int S_{1}a\left(\theta_{k},\phi_{k}\right)s_{k}\left(t\right)\times \rho_{k}\left(\theta_{k},\phi_{k};\mu_{k}\right)d\theta d\phi +n_{1}\left(t\right)\in C^{\tilde{M}\times M}\\ x_{CD2}\left(t\right)=\sum_{k=1}^{K_{CD}}\int \int S_{2}a\left(\theta_{k},\phi_{k}\right)s_{k}\left(t\right)\times \rho_{k}\left(\theta_{k},\phi_{k};\mu_{k}\right)d\theta d\phi +n_{2}\left(t\right)\in C^{\tilde{M}\times M}\\ x_{CD3}\left(t\right)=\sum_{k=1}^{K_{CD}}\int \int S_{3}a\left(\theta_{k},\phi_{k}\right)s_{k}\left(t\right)\times \rho_{k}\left(\theta_{k},\phi_{k};\mu_{k}\right)d\theta d\phi +n_{3}\left(t\right)\in C^{\tilde{M}\times M} \end{array}$$

where $n_{1},n_{2},n_{3}$ are additive noise vectors and $s_{k}\left(t\right)$ is a random process of the *k*-th signal source.

The generalized steering vectors [30] of the *k*-th CD source belonging to three sub-arrays can be respectively expressed as:

(42) $$\begin{array}{cc} b_{CD1}\left(\theta_{k},\phi_{k};\mu_{k}\right)= & \int \int S_{1}a\left(\theta_{k},\phi_{k}\right)\rho_{k}\left(\theta_{k},\phi_{k};\mu_{k}\right)d\theta d\phi \in C^{\tilde{M}\times 1}\\ b_{CD2}\left(\theta_{k},\phi_{k};\mu_{k}\right)= & \int \int S_{2}a\left(\theta_{k},\phi_{k}\right)\rho_{k}\left(\theta_{k},\phi_{k};\mu_{k}\right)d\theta d\phi \in C^{\tilde{M}\times 1}\\ b_{CD3}\left(\theta_{k},\phi_{k};\mu_{k}\right)= & \int \int S_{3}a\left(\theta_{k},\phi_{k}\right)\rho_{k}\left(\theta_{k},\phi_{k};\mu_{k}\right)d\theta d\phi \in C^{\tilde{M}\times 1} \end{array}$$

where $k=1,\ldots ,K_{CD}$.

**Proposition** **2.**  *For a small angular extension, there is an approximate rotational invariant relationships between* $b_{CD1}\left(\theta_{k},\phi_{k};\mu_{k}\right)$ *and* $b_{CD2}\left(\theta_{k},\phi_{k};\mu_{k}\right)$,

(43) $$\begin{array}{cc} b_{CD2}\left(\theta_{k},\phi_{k};\mu_{k}\right)= & F_{2}\left(\theta_{k},\phi_{k}\right)b_{CD1}\left(\theta_{k},\phi_{k};\mu_{k}\right)\\ b_{CD3}\left(\theta_{k},\phi_{k};\mu_{k}\right)= & F_{3}\left(\theta_{k},\phi_{k}\right)b_{CD1}\left(\theta_{k},\phi_{k};\mu_{k}\right) \end{array}$$

*where* $k=1,\ldots ,K_{CD}$.

**Proof.**  see Appendix of [30]. ☐

Then, we can rewrite them in matrix form as:

(44) $$\begin{array}{c} B_{CD2}\approx B_{ID1}W_{x}\\ B_{CD3}\approx B_{ID1}W_{y} \end{array}$$

where:

(45) $$\begin{array}{c} B_{CD1}=\left[b_{CD1}\left(\theta_{k},\phi_{k};\mu_{k}\right),\ldots ,\right.\left.b_{CD1}\left(\theta_{K_{CD}},\phi_{K_{CD}};\mu_{K_{CD}}\right)\right]\in C^{\tilde{M}\times K_{CD}} \end{array}$$

(46) $$\begin{array}{c} B_{CD2}=\left[b_{CD2}\left(\theta_{k},\phi_{k};\mu_{k}\right),\ldots ,\right.\left.b_{CD2}\left(\theta_{K_{CD}},\phi_{K_{CD}};\mu_{K_{\mathrm{CD}}}\right)\right]\in C^{\tilde{M}\times K_{CD}} \end{array}$$

(47) $$\begin{array}{c} B_{CD3}=\left[b_{CD3}\left(\theta_{k},\phi_{k};\mu_{k}\right),\ldots ,\right.\left.b_{CD3}\left(\theta_{K_{CD}},\phi_{K_{CD}};\mu_{K_{CD}}\right)\right]\in C^{\tilde{M}\times K_{CD}} \end{array}$$

(48) $$\begin{array}{c} W_{x}=\text{diag}\left[F_{2}\left(\theta_{1},\phi_{1}\right),\ldots ,\right.\left.F_{2}\left(\theta_{K_{CD}},\phi_{K_{CD}}\right)\right]\in C^{K_{CD}\times K_{CD}} \end{array}$$

(49) $$\begin{array}{c} W_{y}=\text{diag}\left[F_{3}\left(\theta_{1},\phi_{1}\right),\ldots ,\right.\left.F_{3}\left(\theta_{K_{CD}},\phi_{K_{CD}}\right)\right]\in C^{K_{CD}\times K_{CD}} \end{array}$$

Based on Algorithm 2, the array manifold matrix for CD sources, which is denoted as ${\hat{A}}_{CD}$, can be estimated. By multiplying the selection matrices in Equation (40), the array manifold matrices of three sub-arrays can be respectively expressed as:

(50) $$\begin{array}{c} {\hat{B}}_{CD1}=S_{1}{\hat{A}}_{CD}\in C^{\tilde{M}\times K_{CD}}\\ {\hat{B}}_{CD2}=S_{2}{\hat{A}}_{CD}\in C^{\tilde{M}\times K_{CD}}\\ {\hat{B}}_{CD3}=S_{3}{\hat{A}}_{CD}\in C^{\tilde{M}\times K_{CD}} \end{array}$$

According to Equation (50), two rotational invariant relationships can be respectively expressed as:

(51) $$\begin{array}{c} {\hat{W}}_{x}={\hat{B}}_{CD1}^{†}{\hat{B}}_{CD2}\\ {\hat{W}}_{y}={\hat{B}}_{CD1}^{†}{\hat{B}}_{CD3} \end{array}$$

The *k*-th diagonal entries of ${\hat{W}}_{x}$ and ${\hat{W}}_{y}$ are defined as $\varepsilon_{xk}$ and $\varepsilon_{yk}$, respectively. The estimators of the nominal 2D DOA $\theta_{k}$ and $\phi_{k}$ for CD sources are respectively given by:

(52) $$\theta_{k}=\arctan\left(\frac{\varepsilon_{xk}}{\varepsilon_{yk}}\right)$$

(53) $${\hat{\phi }}_{k}=\arcsin\left(\frac{1}{u}\sqrt{-{\left[\ln^{2}\left(\varepsilon_{xk}\right)+\ln^{2}\left(\varepsilon_{yk}\right)\right]}^{2}}\right)$$

where $k=1,\ldots ,K_{CD}$. Thus, the 2D DOA estimation for CD sources are completed. The pseudo-code is summarized as Algorithm 4.
**Algorithm 4** The DOA Estimation Algorithm for CD Sources.
1:Define three $\tilde{M}\times M$ selection matrices according to Equation (40);2:Based on Algorithm 2, the array manifold matrix ${\hat{A}}_{CD}$ for CD sources is estimated;3:Divide the URA into three sub-arrays; the array manifold matrix of these can be obtained by multiplying the corresponding selection matrix based on Equation (50);4:Calculate the two rotational invariant relationships, ${\hat{W}}_{x}$ and ${\hat{W}}_{y}$, according to Equation (51);5:Estimate 2D DOA for CD sources according to Equations (52) and (53).


Thus, the 2D DOA estimation for ID and CD sources can be summarized as follows.
**Algorithm 5** The DOA Estimation Algorithm for Mixed Sources.
1:Separate the ID and CD sources based on SOBI algorithms Algorithm 1 and Algorithm 2;2:Estimate the 2D DOA for ID sources according to Algorithm 3;3:Estimate the 2D DOA for CD sources according to Algorithm 4;


Remark: The proposed algorithm can estimate 2D DOAs of ID or CD sources or joint ID and CD sources. If only ID or CD sources exist in the incident signals, the separation of ID and CD sources can be avoided. It is worth noting that the part of the proposed approach for 2D DOA estimation of ID sources can also be applied to other scenarios by exploiting the rotational invariance property of the array’s structure, such as uniform linear arrays (ULAs) and uniform cylindrical arrays (UCyA) [13]. The part of the proposed approach for 2D-DOA estimation of the CD source has a similar characteristic, *i.e.*, the rotational invariance property of the antenna array’s structure is exploited. Thus, the proposed approach can be applied to other scenarios by exploiting the rotational invariance property of the array’s structure, *i.e.*, there exist three sub-arrays (the array’s structure can be arbitrary), which can construct two different rotational invariance relationships; the 2D DOA estimation for ID and CD sources can be achieved with little modification of the proposed algorithm.

## 5. Simulation Results

In this section, we will illustrate the performance of the proposed algorithm. The MUSIC-based method is used after source separation. The PM [30], MUSIC-based algorithm [31] and the CRB are considered for the performance comparison. In the simulation, the proposed algorithm is called the ESPRIT-based algorithm.

The simulation condition is introduced as follows. The snapshot number is T=1000. The group number of the sampling covariance matrix is L=10 in the SOBI algorithm. The numbers of the elements are 100, *i.e.*, there are $M_{x}=10$ and $M_{y}=10$ in the *x*-axis and *y*-axis, respectively. The number of biosensors is K=5, *i.e.*, one ID source and one CD source. The number of multipaths for the ID source is N=50. The nominal azimuth and elevation for the ID source are 20°, 30°, 30°, 50°, respectively. The azimuth and elevation angular spreads for ID source are both equal to $\sigma_{\vartheta_{ID}}=\sigma_{\phi_{ID}}=\;1^{\circ }$. For the CD source, the nominal azimuth and elevation for the CD source are 60°, 40°, 70°, 50° and 80°, 40°, respectively. The azimuth and elevation angular spreads for the CD source are both equal to $\sigma_{\vartheta_{2}}=\sigma_{\phi_{2}}=2^{\circ }$. The ID source and the CD source both satisfy the Gaussian-shaped distribution. The noise is the additive Gaussian white noise, and it is not correlated with signals. Five hundred Monte Carlo trials are taken in the simulation. The average root mean square error (RMSE) is defined as:

(54) $$RMSE=\sqrt{\frac{1}{500}\sum_{m=1}^{500}{\left({\hat{\kappa }}_{k}\left(m\right)-\kappa_{k}\right)}^{2}}$$

where *κ* stands for ${\hat{\theta }}_{k}\left(m\right)$ and ${\hat{\phi }}_{k}\left(m\right)$, which are the estimates of $\theta_{k}$ and $\phi_{k}$ of the *m*-th Monte Carlo trials, respectively.

**Figure 5:**
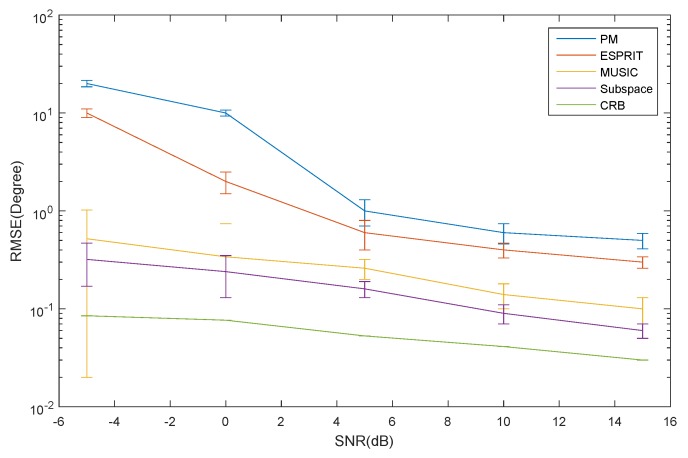
The RMSE of azimuth for the ID source *versus* SNR.

As shown in Figure 5, the RMSE of the azimuth estimation for the ID source *versus* SNR is depicted. It can be seen that the RMSE of the ESPRIT algorithm is larger than that of the MUSIC algorithm, and the CRB has a low SNR. When the SNR increases, the RMSE of the ESPRIT algorithm decrease rapidly. However, the MUSIC algorithm varies slowly as the SNR changes. The PM has the largest RMSE of all of the algorithms. This is mainly caused by the signal and noise subspaces not being orthogonal. The subspace method has the lowest RMSE. However, the computational complexity is tremendous.

**Figure 6:**
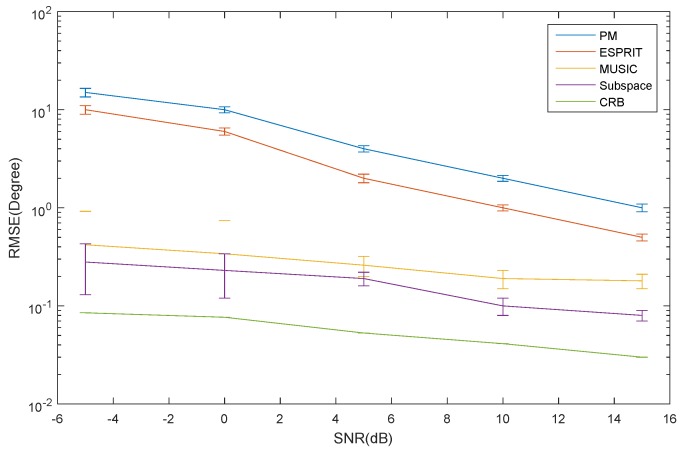
The RMSE of the elevation for the ID source *versus* SNR.

**Figure 7:**
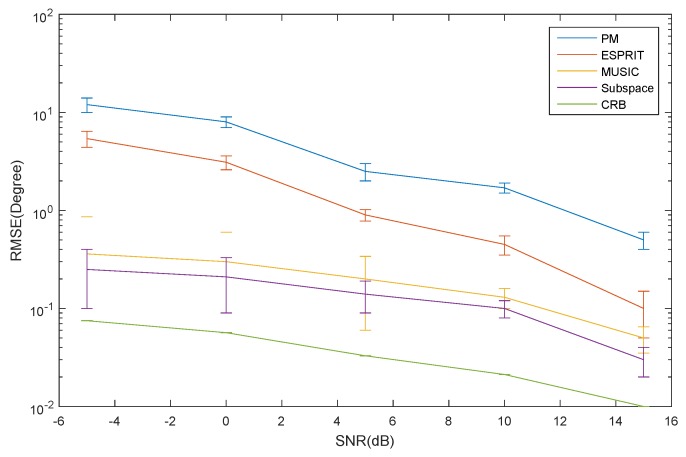
The RMSE of the azimuth for the CD source *versus* SNR.

**Figure 8:**
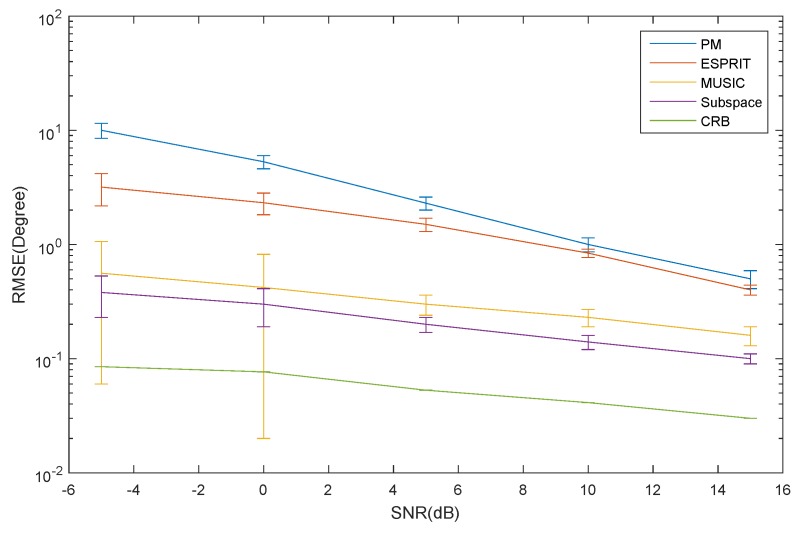
The RMSE of the elevation for the CD source *versus* SNR.

The RMSE of the elevation estimation for the ID source *versus* SNR is depicted in Figure 6. The RMSE of the azimuth and elevation estimation for the CD source *versus* SNR is depicted in Figure 7 and Figure 8, respectively. The curve trend of the PM, the ESPRIT algorithm, the MUSIC and subspace algorithm and the CRB in Figure 6, Figure 7 and Figure 8 are similar. It can be seen that when SNR is low, the RMSE of the ESPRIT algorithm is larger than that of MUSIC, the subspace algorithms and the CRB. The RMSE of the MUSIC and subspace algorithms become smaller as the SNR increases, but the reduced amplitude is not obvious.

**Figure 9:**
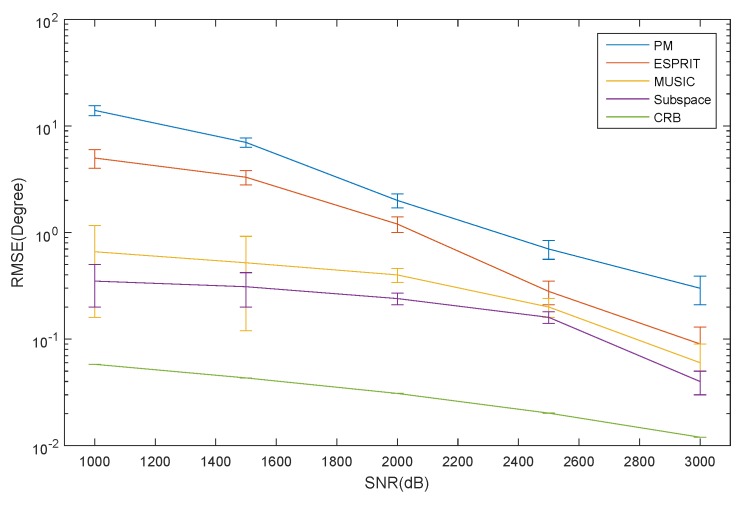
The RMSE of azimuth for the ID source *versus* snapshot number.

As shown in Figure 9, the RMSE of the azimuth estimation for ID source *versus* snapshot number is depicted. It can be seen that the RMSE of the ESPRIT algorithm is larger than that of the MUSIC and subspace algorithms.

**Figure 10:**
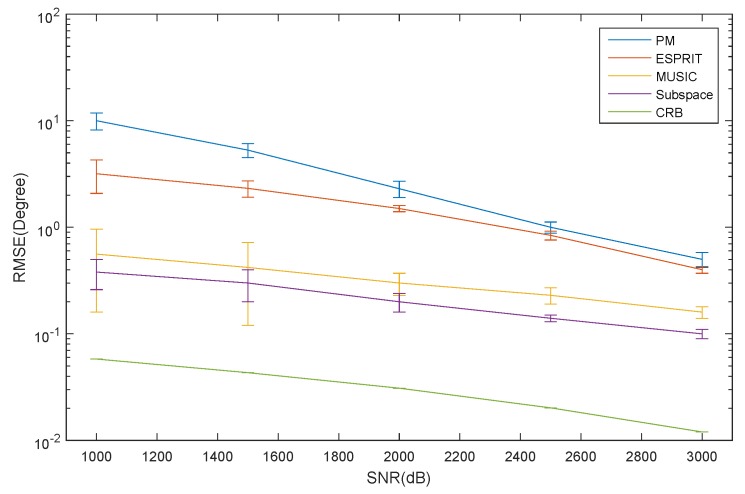
The RMSE of elevation for the ID source *versus* snapshot number.

**Figure 11:**
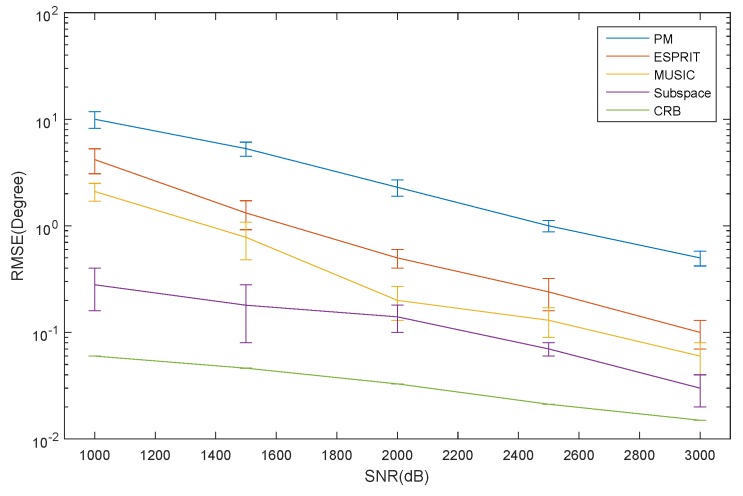
The RMSE of azimuth for the CD source *versus* snapshot number.

**Figure 12:**
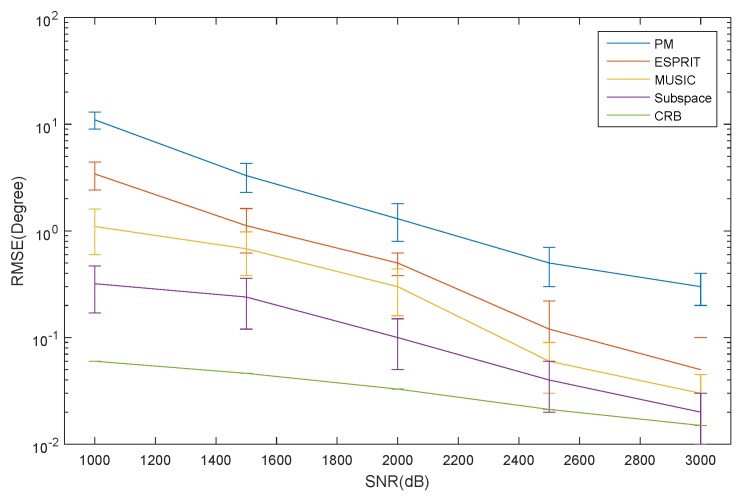
The RMSE of elevation for the CD source *versus* snapshot number.

The RMSE of the elevation estimation for the ID source *versus* snapshot number from UTs is depicted in Figure 10. The RMSE of the azimuth and elevation estimation for the CD source *versus* snapshot number is depicted in Figure 11 and Figure 12, respectively. The curve trends of the PM, ESPRIT, MUSIC and subspace algorithms and the CRB in Figure 10, Figure 11 and Figure 12 are similar, as well. It can be seen that the RMSE of the ESPRIT algorithm is larger than that of the MUSIC and subspace algorithms and the CRB.

We evaluate the averaged CPU times of PM, MUSIC, the subspace algorithm and ESPRIT in the following experiment. The simulation condition is the same as Figure 1. The snapshot number is fixed at 2000, and the SNR is fixed at 3 dB. The experiment is carried out in MATLAB v.8.3.0 on a PC with a Windows 7 system and a 3-GHz CPU. Table 1 presents the the averaged CPU times of PM, MUSIC, the subspace algorithm and ESPRIT. The MUSIC and subspace method is the most time consuming, since the spectrum peak searching is needed. PM costs the least time of all, since the spectrum peak searching is not needed. Thus, ESPRIT is chosen as the DOA estimation algorithm for the biosensor’s localization, since it performs well in the estimated accuracy and resolution probability, and it does not cost much time to execute the algorithm.

It should be noted that the curve of the MUSIC algorithm does not change much in the above figure. This phenomenon is mainly caused by the MUSIC algorithm being directly used by multiplying the estimated array manifold matrix without any form transformation.

The proposed algorithm does not perform well when the SNR is low. However, as we all know, the BS of VMIMO is equipped with a large number of elements. We can deal with this problem by increasing the number of elements equipped in the BS as a trade-off. The proposed algorithm has a larger RMSE than that of the MUSIC algorithm, but the proposed algorithm has much lower computational complexity compared to the MUSIC algorithm.

## 6. Conclusions

For the location of the biosensor (wearable sensor) in health monitoring systems, first, a localization scheme based on a multiple VMIMO system is constructed. The intersecting point of two rays, which come from two different directions measured by two uniform rectangle arrays (URA), can be regarded as the location of the biosensor (wearable sensor). Then, a 2D DOA estimation algorithm under the coexistence of ID and CD sources is proposed. The computational complexity of the proposed algorithm is much lower than that of other multidimensional parameter searching algorithms, such as the MUSIC algorithm. The ID and CD sources are processed separately. Three sub-arrays are selected to construct rotational-invariant matrices. The simulation result confirms that the proposed algorithm outperforms the MUSIC-based algorithm when the SNR is larger than a certain threshold value. In future work, we will focus on the effective separation method between the ID and CD sources with low computational complexity. In addition, the angular spreads’ estimation should be considered in our future work.

## Figures and Tables

**Table 1:** Averaged CPU times. Time unit: s.

PM	ESPRIT	MUSIC	Subspace
0.28	0.61	1.20	2.5

**Figure A1:**
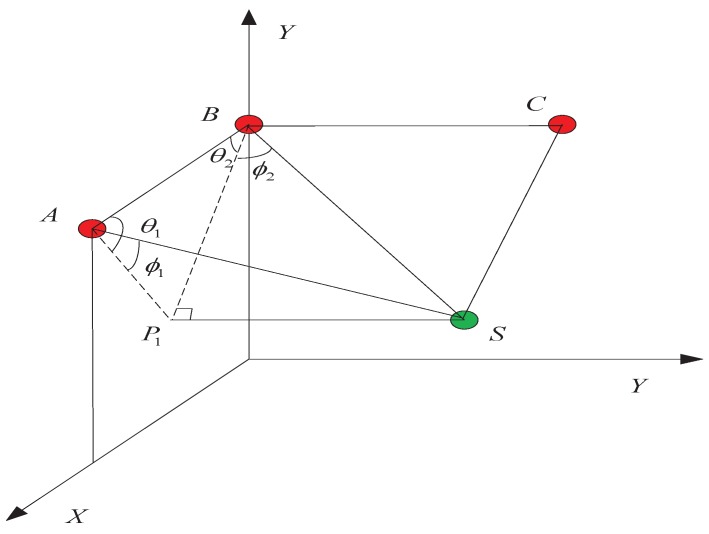
The localization of the biosensor.

## Appendix A

### A1. The Localization Method Based on Multiple VMIMO

A similar scheme has been proposed in our previous work [32,33]. It is used for the localization of patients in the emergency healthcare system and partial-discharge source diagnosis and localization in an industrial high-voltage insulation system. The effectiveness of the proposed scheme in [32,33] has been verified. The real test has been done for partial-discharge source localization [33]. In order to have an intuitive impression, we give the localization method of the proposed scheme as follows.

As shown in Figure A1, three URAs cooperate with each other to locate the biosensors’ positions. The azimuths $\theta_{1}$, $\phi_{1}$ and $\theta_{2}$, $\phi_{2}$ can be estimated by using the algorithm proposed in Section 4. The coordinates of reference Points A, B and C corresponding to VMIMO1(URA1), VMIMO2(URA2) and VMIMO3(URA3) are $\left(x_{A},0,z\right)$, $\left(0,0,z\right)$ and $\left(0,y_{C},0\right)$, respectively. Based on the measurements of URA1 and URA2, we have:

(A1) $$x_{s}=\frac{x_{A}}{\frac{\tan\theta_{2}}{\tan\theta_{1}}+1}$$

(A2) $$y_{s}=\frac{x_{A}}{\tan^{-1}\theta_{1}+\tan^{-1}\theta_{2}}\sqrt{1+\tan^{-2}\theta_{1}}\tan\phi_{1}$$

(A3) $$z_{s}=z-\frac{x_{A}}{\tan^{-1}\theta_{1}+\tan^{-1}\theta_{2}}$$

Based on the measurements of URA2 and URA3, we would have another estimated result. These two results can be used to improve the estimated stability of the proposed scheme.

### A2. The Derivation of Equation (9)

Assume the *k*-th ID source impinges on the array; the covariance matrix of received data can be modeled as:

(A4) $$\begin{array}{cc} R_{IDk}= & 2\pi S_{IDk}\int \int \int \int \left\{p\left(\alpha \right)\right.a_{k}\left(\theta_{i},\phi_{i}\right)a_{k}^{H}\left(\theta_{j},\phi_{j}\right)\\ & \times \left.q_{k}\left(\left(\theta_{i},\phi_{i}\right),\left(\theta_{j},\phi_{j}\right);\sigma_{\theta },\sigma_{\phi }\right)\right\}d\theta_{i}d\phi_{i}d\theta_{j}d\phi_{j}+\sigma_{n}^{2}I \end{array}$$

where $q_{k}\left(\left(\theta_{i},\phi_{i}\right),\left(\theta_{j},\phi_{j}\right);\sigma_{\theta },\sigma_{\phi }\right)$ is the kernel function of angular auto-correlation. $p\left(\alpha \right)$ is the von Mises angular function, whose definition is:

(A5) pα=expαcosθi,ϕi−θj,ϕjexpαcosθi,ϕi−θj,ϕj2πJ0α2πJ0α

where $J_{0}\left(\alpha \right)$ is first kind zero-order Bessel function, whose definition is:

(A6) J0α=∑n=0∞kk222ikk222in!2n!2

When α=∞, we have $p\left(\alpha \right)=\delta \left(\left(\theta_{i},\phi_{i}\right)-\left(\theta_{j},\phi_{j}\right)\right)$. If $\sigma_{\theta }>0$ and $\sigma_{\phi }>0$, we have:

(A7) $$\begin{array}{c} R_{IDk}=S_{IDk}\int \int a_{k}\left(\theta ,\phi \right)a_{k}^{H}\left(\theta ,\phi \right)p_{I}\left(\theta ,\phi ,\sigma_{\theta },\sigma_{\phi }\right)d\theta d\phi \\ \approx S_{IDk}a_{k}\left(\theta ,\phi \right)a_{k}^{H}\left(\theta ,\phi \right)⊙B\left(\Phi_{k}\right) \end{array}$$

where $p_{I}\left(\theta ,\phi ,\sigma_{\theta },\sigma_{\phi }\right)$ is the angular power density of the ID source and $B\left(\Phi_{k}\right)$ is a real-valued symmetric Toeplitz matrix.

### A3. The Proof of Proposition 1

Two $\left(a-1\right)\times a$ selection matrices $J_{a\times 0}=\left[I_{a-1},0_{\left(a-1\right)\times 1}\right]$ and $J_{a\times 1}=\left[0_{\left(a-1\right)\times 1},I_{a-1}\right]$ are defined, where $0_{a\times b}$ is an a×b zero matrix. Thus, the steering vector of Sub-array 1, Sub-array 2 and Sub-array 3 can be respectively expressed as:

(A8) $$b_{ID1}=\left(J_{M_{y}\times 0}a_{y}\right)⊗\left(J_{M_{x}\times 0}a_{x}\right)$$

(A9) $$b_{ID2}=\left(J_{M_{y}\times 1}a_{y}\right)⊗\left(J_{M_{x}\times 0}a_{x}\right)$$

(A10) $$b_{ID3}=\left(J_{M_{y}\times 0}a_{y}\right)⊗\left(J_{M_{x}\times 1}a_{x}\right)$$

From Equations (A8)–(A10), we can obtain the relationships among the steering vector of sub-arrays:

(A11) $$b_{IDq}\left({\bar{\theta }}_{k},{\bar{\phi }}_{k}\right)=F_{q}\left({\bar{\theta }}_{k},{\bar{\phi }}_{k}\right)b_{ID1}\left({\bar{\theta }}_{k},{\bar{\phi }}_{k}\right)$$

where q=2,3 and:

(A12) $$F_{2}\left({\bar{\theta }}_{k},{\bar{\phi }}_{k}\right)=\exp\left(i\omega \sin\left({\bar{\phi }}_{k}\right)\cos\left({\bar{\theta }}_{k}\right)\right)$$

(A13) $$F_{3}\left({\bar{\theta }}_{k},{\bar{\phi }}_{k}\right)=\exp\left(i\omega \sin\left({\bar{\phi }}_{k}\right)\sin\left({\bar{\theta }}_{k}\right)\right)$$

where $F_{2}\left({\bar{\theta }}_{k},{\bar{\phi }}_{k}\right)$ and $F_{3}\left({\bar{\theta }}_{k},{\bar{\phi }}_{k}\right)$ are two different rotational-invariant relationships containing the information of 2D nominal DOAs. Then, the partial derivation of $b_{IDq}\left({\bar{\theta }}_{k},{\bar{\phi }}_{k}\right)$ can be respectively expressed using $b_{1}\left({\bar{\theta }}_{k},{\bar{\phi }}_{k}\right)$ and its partial derivation as:

(A14) $$\frac{\partial b_{IDq}\left({\bar{\theta }}_{k},{\bar{\phi }}_{k}\right)}{\partial \theta }=F_{q}\left({\bar{\theta }}_{k},{\bar{\phi }}_{k}\right)\frac{\partial b_{ID1}\left({\bar{\theta }}_{k},{\bar{\phi }}_{k}\right)}{\partial \theta }+\frac{\partial F_{q}\left({\bar{\theta }}_{k},{\bar{\phi }}_{k}\right)}{\partial \theta }b_{ID1}\left({\bar{\theta }}_{k},{\bar{\phi }}_{k}\right)$$

and:

(A15) $$\frac{\partial b_{IDq}\left({\bar{\theta }}_{k},{\bar{\phi }}_{k}\right)}{\partial \phi }=F_{q}\left({\bar{\theta }}_{k},{\bar{\phi }}_{k}\right)\frac{\partial b_{ID1}\left({\bar{\theta }}_{k},{\bar{\phi }}_{k}\right)}{\partial \phi }+\frac{\partial F_{q}\left({\bar{\theta }}_{k},{\bar{\phi }}_{k}\right)}{\partial \phi }b_{ID1}\left({\bar{\theta }}_{k},{\bar{\phi }}_{k}\right)$$

Based on Equation (18), the array manifold matrix of the *q*-th sub-array can be expressed as:

(A16) $$\begin{array}{cc} B_{IDl}= & \left[a_{l}\left({\bar{\theta }}_{1},{\bar{\phi }}_{1}\right),\ldots ,a_{l}\left({\bar{\theta }}_{K_{ID}},{\bar{\phi }}_{K_{ID}}\right)\right.,\frac{\partial a_{l}\left({\bar{\theta }}_{1},{\bar{\phi }}_{1}\right)}{\partial {\bar{\theta }}_{1}}\\ & ,\ldots ,\frac{\partial a_{l}\left({\bar{\theta }}_{K_{ID}},{\bar{\phi }}_{K_{ID}}\right)}{\partial {\bar{\theta }}_{K_{ID}}},\left.\frac{\partial a_{l}\left({\bar{\theta }}_{1},{\bar{\phi }}_{1}\right)}{\partial {\bar{\phi }}_{1}},\ldots ,\frac{\partial a_{l}\left({\bar{\theta }}_{K_{ID}},{\bar{\phi }}_{K_{ID}}\right)}{\partial {\bar{\phi }}_{K_{ID}}}\right] \end{array}$$

Then, there exist two rotational invariant matrices $W_{q,1}$ satisfying $B_{ID1}W_{q,1}=B_{IDq}$, where:

(A17) $$W_{q,1}=\left[\begin{array}{ccc} P_{q,1} & P_{q,2} & P_{q,3}\\ 0_{K_{ID}\times K_{ID}} & P_{q,1} & 0_{K_{ID}\times K_{ID}}\\ 0_{K_{ID}\times K_{ID}} & 0_{K_{ID}\times K_{ID}} & P_{q,1} \end{array}\right]\in C^{3K_{ID}\times 3K_{ID}}$$

(A18) $$P_{q,1}=\text{diag}\left[F_{q}\left({\bar{\theta }}_{1},{\bar{\phi }}_{1}\right),\ldots ,F_{q}\left({\bar{\theta }}_{K},{\bar{\phi }}_{K}\right)\right]\in C^{3K_{ID}\times 3K_{ID}}$$

(A19) $$P_{q,2}=\text{diag}\left[\frac{\partial F_{q}\left({\bar{\theta }}_{1},{\bar{\phi }}_{1}\right)}{\partial {\bar{\theta }}_{1}},\ldots ,\frac{\partial F_{q}\left({\bar{\theta }}_{1},{\bar{\phi }}_{1}\right)}{\partial {\bar{\theta }}_{1}}\right]\in C^{3K_{ID}\times 3K_{ID}}$$

(A20) $$P_{q,3}=\text{diag}\left[\frac{\partial F_{q}\left({\bar{\theta }}_{1},{\bar{\phi }}_{1}\right)}{\partial {\bar{\phi }}_{1}},\ldots ,\frac{\partial F_{q}\left({\bar{\theta }}_{1},{\bar{\phi }}_{1}\right)}{\partial {\bar{\phi }}_{1}}\right]\in C^{3K_{ID}\times 3K_{ID}},q=2,3$$

Based on Equations (A14), (A15) and (A17), we can know that diagonal entries of $W_{q,1}$ contain the 2D nominal DOAs:

(A21) $${\left[W_{2,1}\right]}_{k+\left(l-1\right)K,k+\left(l-1\right)K}=F_{2}\left({\bar{\theta }}_{k},{\bar{\phi }}_{k}\right)$$

(A22) $${\left[W_{3,1}\right]}_{k+\left(l-1\right)K,k+\left(l-1\right)K}=F_{3}\left({\bar{\theta }}_{k},{\bar{\phi }}_{k}\right)$$

where l=1,2,3. Thus, diagonal entries of $W_{q,1}$ can be used for estimating the 2D nominal DOAs.

From Equation (25), we can obtain the signal subspace $U_{IDl}$ corresponding to the array manifold matrix of the *l*-th sub-array:

(A23) $$B_{IDl}=U_{IDl}T,l=1,2,3$$

Because 2D nominal DOAs’ information is contained in $W_{q,1}$, the relationship $B_{ID1}W_{q,1}=B_{IDq}$ can be used to estimate DOA, and they are substituted in Equation (A20); we have:

(A24) $$\begin{array}{c} B_{ID1}=U_{ID1}T\\ B_{ID1}W_{2,1}=U_{ID2}T\\ B_{ID1}W_{3,1}=U_{ID3}T \end{array}$$

Based on the ESPRIT algorithm, we have Equation (28). Thus, the proof is completed.

### A4. Cramér-Rao Bound

In this section, the CRB of 2D DOA estimation, including ID and CD sources, is derived. Generally, the received data of URA can be expressed as:

(A25) $$x\left(t\right)=\sum_{k=1}^{K}s_{k}\left(t\right)h_{k}\left(t\right)=Hs\left(t\right)$$

where $x\left(t\right)$, a complex Gaussian vector with zero mean, $h_{k}\left(t\right)$ is the *k*-th channel parameter, $H\in C^{M\times K}$ is the channel parameter matrix and $s\left(t\right)\in C^{K\times 1}$ is the signal vector. The covariance matrix of $x\left(t\right)$ is expressed as:

(A26) $$R_{x}=E\left\{x\left(t\right)x{\left(t\right)}^{H}\right\}=HR_{s}H^{H}$$

For *T* statistically-independent observations of $x\left(t\right)$, the unknown DOAs vector of $R_{x}$ is defined as:

(A27) $$\kappa ={\left[\theta^{T},\phi^{T}\right]}^{T}$$

where $\theta ={\left[\theta_{1},\ldots ,\theta_{K_{ID}},\theta_{K_{ID}+1},\ldots ,\theta_{K}\right]}^{T}$ and $\phi ={\left[\phi_{1},\ldots ,\phi_{K_{ID}},\phi_{K_{ID}+1},\ldots ,\phi_{K}\right]}^{T}$. The general expression of the $\left(m,n\right)$-th element in the Fisher information matrix (FIM) can be expressed as:

(A28) $$F_{\kappa_{m}\kappa_{n}}=Ttr\left\{R_{x}^{-1}\frac{\partial R_{x}}{\partial \kappa_{m}}R_{x}^{-1}\frac{\partial R_{x}}{\partial \kappa_{n}}\right\}$$

In the following derivation, H˙ϑm=Δ∂H∂H∂ϑm∂ϑm. Based on Equation (A26), the partial derivation of $R_{x}$ is:

(A29) $$\frac{\partial R_{x}}{\partial \theta_{m}}={\dot{H}}_{\vartheta_{m}}R_{s}H^{H}+HR_{s}{\dot{H}}_{\vartheta_{m}}^{H}$$

Based on trR+RH=2RetrR, we have:

(A30) $$\begin{array}{cc} F_{\theta_{m}\theta_{n}}= & Ttr\left\{R_{x}^{-1}\frac{\partial R_{x}}{\partial \theta_{m}}R_{x}^{-1}\frac{\partial R_{x}}{\partial \theta_{n}}\right\}\\ = & Ttr\left\{R_{x}^{-1}\left({\dot{H}}_{\vartheta_{m}}R_{s}H^{H}+HR_{s}{\dot{H}}_{\vartheta_{m}}^{H}\right)R_{x}^{-1}\right.\left.\left({\dot{H}}_{\vartheta_{n}}R_{s}H^{H}+HR_{s}{\dot{H}}_{\vartheta_{n}}^{H}\right)\right\}\\ = & 2TRe\left\{tr\left\{R_{x}^{-1}{\dot{H}}_{\vartheta_{m}}R_{s}H^{H}R_{x}^{-1}{\dot{H}}_{\vartheta_{n}}R_{s}H^{H}\right\}tr\left\{R_{x}^{-1}{\dot{H}}_{\vartheta_{m}}R_{s}H^{H}R_{x}^{-1}HR_{s}{\dot{H}}_{\vartheta_{n}}^{H}\right\}\right\} \end{array}$$

Since only the *m*-th column of ${\dot{H}}_{\vartheta_{m}}$ is nonzero, then it can be represented as ${\dot{H}}_{\vartheta_{m}}=H_{\theta }D_{K}^{m}{\left(D_{K}^{m}\right)}^{T}$, where the *m*-th column of the identity matrix is defined as $\gamma_{K}^{m}$. $H_{\theta }$ is the derivative matrix of the channel parameter matrix, which is expressed as:

(A31) $$H_{\theta }=\left[\frac{dh_{1}}{d\theta_{1}},\ldots ,\frac{dh_{K_{ID}}}{d\theta_{K_{ID}}},\frac{dh_{K_{ID}+1}}{d\theta_{K_{ID}+1}},\ldots ,\frac{dh_{K}}{d\theta_{K}}\right]$$

Then, Equation (A30) can be rewritten as:

(A32) Fθmθn=2TRetrRx−1HθDKmDKmTRsHHRx−1HθDKnDKnTRsHH+Rx−1HRsDKmDKmTHθHRx−1HθDKnγKnTRsHH=2TReDKmTRsHHRx−1HθDKnDKnTRsHHRx−1HθDKm+DKmTHθHRx−1HθDKnDKnTRsHHRx−1HRsDKm

The FIM corresponding to ***θ*** can be expressed as:

(A33) Fθθ=2TReRsHHRx−1Hθ⊙RsHHRx−1HθT+HθHRx−1Hθ⊙RsHHRx−1HRsT

Similarly, the FIM corresponding to ***ϕ*** can be expressed as:

(A34) Fϕϕ=2TReRsHHRx−1Hϕ⊙RsHHRx−1HϕT+HϕHRx−1Hϕ⊙RsHHRx−1HRsT

where:

(A35) $$H_{\phi }=\left[\frac{dh_{1}}{d\phi_{1}},\ldots ,\frac{dh_{K_{ID}}}{d\phi_{K_{ID}}},\frac{dh_{K_{ID}+1}}{d\phi_{K_{ID}+1}},\ldots ,\frac{dh_{K}}{d\phi_{K}}\right]$$

The FIM corresponding to the cross-terms between ***θ*** and ***ϕ*** are respectively expressed as:

(A36) Fθϕ=2TReRsHHRx−1Hϕ⊙RsHHRx−1HθT+HθHRx−1Hϕ⊙RsHHRx−1HRsT

and:

(A37) Fϕθ=2TReRsHHRx−1Hθ⊙RsHHRx−1HϕT+HϕHRx−1Hθ⊙RsHHRx−1HRsT

Based on the derivation above, the entire FIM can be expressed as:

(A38) $$F_{\kappa \kappa }={\left[\begin{array}{cc} F_{\theta \theta } & F_{\theta \phi }\\ F_{\phi \theta } & F_{\phi \phi } \end{array}\right]}_{2K\times 2K}$$

Define a new matrix $G=F_{\kappa \kappa }^{-1}$; we can obtain the CRBs of *θ* and *ϕ* for the ID and CD sources, respectively, as:

(A39) CRBθID=∑k=1KIDGkkGkkKIDKID

(A40) CRBθCD=∑k=KID+1KGkkGkkKCDKCD

(A41) CRBϕID=∑k=K+1K+KIDGkkGkkKIDKID

(A42) CRBϕCD=∑k=K+KID+12KGkkGkkKCDKCD
